# Extracellular Vesicles Derived From the Feces of Pregnant Women Modulate T Cells Toward a Pregnancy‐Supportive Phenotype In Vitro

**DOI:** 10.1002/eji.70056

**Published:** 2025-09-19

**Authors:** Stefanie Dietz‐Ziegler, Samantha Kewitz, Gabriele Kaiser, Jessica Rühle, Alexander Marmé, Alexander Dalpke, Bachar Cheaib, Jan Pauluschke‐Fröhlich, Melanie Henes, Ana Velic, Andreas Pich, Anneli Vollert, Martin Schaller, Felix Knab, Trim Lajqi, Christian F. Poets, Christian Gille, Natascha Köstlin‐Gille

**Affiliations:** ^1^ Department of Neonatology Tuebingen University Children's Hospital Tuebingen Germany; ^2^ Department of Neonatology Heidelberg University Children's Hospital Heidelberg Germany; ^3^ Gynecology and Obstetrics Practice Am Lustnauer Tor Tuebingen Germany; ^4^ Department of Infectious Diseases Medical Microbiology and Hygiene, Medical Faculty Heidelberg University Heidelberg Germany; ^5^ Translational Lung Research Center (TLRC) Heidelberg German Center for Lung Research (DZL) Heidelberg University Heidelberg Germany; ^6^ Department of Women's Health University of Tuebingen Tuebingen Germany; ^7^ Interfaculty Institute for Cell Biology Proteome Center Tuebingen (PCT) University of Tuebingen Tuebingen Germany; ^8^ Core Facility Proteomics Hannover Medical School Institute of Toxicology Hannover Germany; ^9^ Department of Dermatology University of Tuebingen Tuebingen Germany; ^10^ Department for Neurodegenerative Diseases Hertie Institute for Clinical Brain Research Tuebingen Germany

**Keywords:** fecal extracellular vesicles, microbiome, pregnancy, T helper cells

## Abstract

Pregnancy requires immune tolerance to a semi‐allogeneic fetus, involving profound adaptations, particularly in the T helper (Th) cell response. The intestinal microbiome plays a crucial role in health, but its influence on immune adaptation to pregnancy remains unclear. Bacterial extracellular vesicles (BEVs), released by gut bacteria, can cross the intestinal barrier and modulate immune responses. In our study we investigated the effect of fecal EVs (fEVs) from pregnant women on Th cell composition *in vitro*. fEVs were purified from preserved stool samples, characterized, and their uptake by immune cells was analyzed. Using an *in vitro* T cell culture model, we examined Th cell phenotypes, intracellular cytokine expression, and proteomic changes after stimulation with fEVs from pregnant and non‐pregnant women. We demonstrate that fEVs from preserved stool samples are rapidly taken up by T cells and modulate their phenotype. Stimulation with fEVs from pregnant women shifts Th cells toward a regulatory profile favorable for pregnancy, increasing Th2 cells while reducing Th17 cells compared to fEVs from non‐pregnant controls. This study provides the first *in vitro* evidence that fecal‐derived EVs influence immune adaptation to pregnancy and may offer a basis for microbiome‐targeted strategies to prevent or treat immunological pregnancy complications.

## Graphical abstract

Fecal extracellular vesicles (fEVs) were isolated from preserved stool samples in DNA/RNA shield and characterized. In an *in vitro* model with PBMC, fEVs of pregnant women modulate T cells toward a regulatory phenotype favorable for pregnancy compared to fEVs of non‐pregnant women.

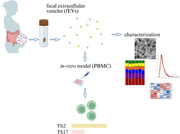

**Abbreviations**
ASV: amplicon sequence variants
CFSE: carboxyfluorescein succinimidyl ester
DC: dendritic cells
EM: electron microscopy
ERK: extracellular‐signal regulated kinases
EVs: extracellular vesicles
FDR: false discovery rate
fEVs: fecal extracellular vesicles
JAK2: Janus kinase 2
LPS: lipopolysaccharide
LTA: lipoteiconic acid
MACS: magnetic activated cell sorting
NTA: nanoparticle tracking analysis
PBMC: peripheral blood mononuclear cells
PE: preeclampsia
PFA: paraformaldehyde
RAS: rat sarcoma
RSU1: Ras suppressor protein 1
STAT5: signal transducer and activator of transcription 5
TEM: transmission electron microscopy
Th cells: T helper cells
T_regs_
: regulatory T cells

## 1 Introduction

During pregnancy, the maternal immune system must be precisely regulated to prevent rejection of the semi‐allogeneic fetus while sustaining an adequate immune response toward infections. A failure of the immunological adaptation processes to pregnancy can lead to severe complications, such as abortions or preeclampsia (PE) [1, 2, 3]. T cells are among the best studied immune cells in pregnancy. Quantities of CD4^+^ T helper (Th) cells and CD8^+^ cytotoxic T cells remain relatively stable [4], but proportions of Th subsets Th1, Th2, Th17, and regulatory T cells (T_regs_) change significantly during pregnancy. Although healthy pregnancy is characterized by a predominance of anti‐inflammatory Th2 responses [5, 6] and an expansion of T_regs_ [7, 8], pregnancy complications such as abortions or PE are associated with increased Th1 and Th17 responses [9, 10, 11, 12, 13, 14].

The intestinal microbiome contributes significantly to maintaining health, but it can also be involved in the pathogenesis of diseases, such as chronic inflammatory bowel disease [15, 16], metabolic syndrome [17, 18], or asthma [19]. During pregnancy, the intestinal microbiome undergoes significant changes similar to those seen in patients with metabolic syndrome, and the transfer of microbiota of third‐trimester pregnant women induced obesity and insulin resistance in mice [20]. However, there is yet little known about how the microbiome influences immune adaptation to pregnancy. One mechanism by which the intestinal microbiome regulates local and systemic inflammation is the release of extracellular vesicles (EVs) by intestinal bacteria [21]. These bacterial nanoparticles are produced by Gram‐positive and Gram‐negative bacteria and carry bacterial molecules, such as lipopolysaccharides (LPS), peptidoglycans, lipids, proteins, and nucleic acids, and in the case of pathogens, toxins and virulence factors [21]. Their high penetrative capacities allow them to distribute widely in the host and to interact with immune cells [22, 23].

Here, we investigated whether fecal EVs (fEVs) have an influence on T cell regulation during pregnancy. To this end, we established a protocol for isolating fEVs from preserved stool samples and investigated the effect of these fEVs on immune cells in an *in vitro* model. We show that fEVs from the stool of pregnant women shift the phenotype of Th cells toward anti‐inflammatory Th2 cells and inhibit pro‐inflammatory Th17 cells, thus changing the Th profile toward a profile favorable for pregnancy. This may be a potential mechanism for a direct influence of the microbiome on the immune system during pregnancy, which could render the microbiome a target for an individualized control of immune processes during pregnancy.

## 2 Methods

### 2.1 Patients

The local ethics committee (Ethics Committee at the Medical Faculty of the Eberhard Karls University and the University Hospital of Tübingen) approved this study (537/2022BO1) and all women gave written informed consent. From May 2022 to March 2023, stool samples from 38 non‐pregnant and pregnant women (aged 23–44 years) were collected. Exclusion criteria for the study were the intake of probiotics, antibiotics, or immune‐modulating therapies in the last 4 weeks. Non‐pregnant control subjects had experienced at least one uncomplicated pregnancy and were not pregnant at the time of study participation. Healthy pregnant women in their second or third trimester of pregnancy were recruited at one co‐author's gynecological practice (AM). Patients suffering from severe pregnancy complications (severe infection, preterm rupture of membranes, preterm labor, (pre‐)eclampsia), chronic diseases (autoimmune diseases, malignancies, chronic infections) or receiving immunosuppressive therapy were excluded. Table S1 shows clinical characteristics of the study participants.

### 2.2 fEV Isolation and Protein Quantification

Stool samples were collected and stored either native or in DNA/RNA shield fecal collection tubes (Zymo Research, Freiburg, Germany) at −80°C. For fEV isolation, stool samples were diluted in PBS to a final concentration of 0.1 g stool in 1 mL PBS. Samples were then divided into aliquots of 600 µL, each aliquot containing 60 mg of stool, from which EVs were isolated according to different protocols (see Table S1). After these protocols had been run, fEVs were purified either by ultracentrifugation (20 h at 36.400 rpm at 4°C) or by precipitation with ExoQuick‐TC (System Biosciences, Palo Alto, USA) according to the manufacturer's protocol. The resulting fEV pellets were stored at −80°C until further use.

### 2.3 Nanoparticle Tracking Analysis (NTA)

To investigate the size distribution and concentration of fEVs, NTA was carried out using a NanoSight NS300 equipped with a sCMOS camera and a laser of 405 nm (Malvern Panalytical, Malvern, UK). Thawed fEV pellets were dissolved in 1000 µL of PBS and loaded into a 1 mL syringe. For each sample, three measurements of 45 s each were performed at a Camera level of 11 and a syringe pump speed of 100 µL/s. Measurements were analyzed with the NanoSight Software NTA 3.4 Build 3.4.003 (Malvern Panalytical) with the detection threshold set at level 5.

### 2.4 Transmission Electron Microscopy (TEM)

fEVs in 2% paraformaldehyde (PFA) were placed directly onto a glow‐discharged electron microscope (EM) grid (formvar/carbon 300 mesh), subsequently contrasted with uranyl acetate and analyzed using a Zeiss LIBRA 120 transmission EM (Zeiss) operating at 120 kV.

### 2.5 CFSE‐Staining of fEVs

To visualize the fEV interaction with T cells, fEVs were stained with CFSE at a final concentration of 5 mM and incubated at 37°C for 1 h. The reaction was blocked by adding EV‐free FCS for 10 min and remaining CFSE was washed out by ultracentrifugation at 36.400 rpm and 4°C for 1 h. The supernatant was discarded and the pellet containing CFSE‐stained fEVs was resuspended in PBS.

### 2.6 Fluorescence Microscopy

1 × 10^6^ Peripheral blood mononuclear cells (PBMCs) were incubated with 1 × 10^9^ CFSE‐stained fEVs for 1 h at 37°C. Free fEVs were washed out by centrifugation at 400 g for 5 min. Cells were then spun onto a coverslip, fixed in 4% PFA, blocked in 5% FBS and incubated over night with CD3 antibody (clone UCHT1, BioLegend, Amsterdam, the Netherlands, diluted 1:500) at 4°C in the dark. The next day, the secondary antibody (anti‐mouse AlexaFluor647, Invitrogen, Schwerte, Germany, diluted 1:400) was incubated for 1 h, nuclei were stained with Dapi (1 µg/µL, Invitrogen). The stained cells on the coverslip were mounted onto a microscope slide and analyzed with an ApoTome System (Zeiss Axio Observer.Z1, Carl Zeiss Microscopy, Oberkochen, Germany).

### 2.7 Isolation of T Cells and Stimulation With fEVs

Human PBMCs were prepared from heparinized blood samples by density gradient centrifugation (Pancoll lymphocyte separation medium, PanBiotech, Aidenbach, Germany). For isolation of T cells from the PBMC fraction, cells were labeled with anti‐CD3‐MicroBeads and separated by magnetic activated cell sorting (MACS) according to the manufacturer's protocol (Miltenyi Biotech, Bergisch Gladbach, Germany).

For stimulation of T cells with fEVs, 1 × 10^6^ T cells were seeded in 1 mL of RPMI medium with 10% FBS, 1% penicillin/streptomycin and 1% glutamine in a 24 well‐plate (Corning Incorporated, Kennebunk, USA). For analysis of fEV uptake by T cells, fEVs (from 20 mg stool) were added to PBMC for 0.25, 0.5, 1, 4, 8, 12 and 24 h and cultured at 37°C and 5% CO_2_. For phenotype and intracellular cytokine expression analyses of T cells after fEV stimulation, T cells/PBMC were stimulated with fEVs in a 1:500 ratio for 24 h. In order to minimize T cell/PBMC donor effects, 11–14 different fEV samples were added to 5 different T cell/PBMC donors.

### 2.8 Flow Cytometry

For quantification of T cell subsets, cells were pre‐gated to CD45, CD3, and CD4. Among CD45^+^/CD3^+^/CD4^+^ cells, cell types were identified as follows: Th1 cells CCR4^−^/CXCR3^+^/CCR6^−^, Th2 cells CCR4^+^/CXCR3^‐^/CCR6^−^, Th17 cells CCR4^+^/CXCR3^‐^/CCR6^+^, T_reg_ CD25^+^ [24–26]. Antibodies used for extracellular staining of human cells were purchased from BioLegend, San Diego, USA (CD45 APC‐Cy7 [clone 2D1, Cat#: 368516], CD3 PE‐Cy7 [clone HIT3a, Cat#: 300316], CCR6 APC [clone G034E3; Cat#: 353415], CD25 BV421 [clone M‐A251, Cat#: 356114]), R&D, Minneapolis, USA (CCR4 FITC [clone 205410, Cat#: FAB1567F] and BD Bioscience, Heidelberg, Germany (CD4 PerCp [clone RPA‐T4, Cat#: 300527], CXCR3 PE [clone 1C6, Cat#: 560928]). Intracellular staining of cytokines was performed as described previously [27]. Antibodies against IL‐4, IL‐17, and IFN‐γ were purchased from BD Biosciences, Heidelberg, Germany (IL‐4 PE clone MP4‐25D2 [Cat#: 559333], IL‐17 PE clone N49‐653 [Cat#: 560486], IFN‐γ PE clone B27 [Cat#: 559327]).

Data acquisition was performed with a FACSCanto II and analyzed via FlowJo V10 (FlowJo, LLC, Ashland, Oregon, USA).

### 2.9 Enzyme‐linked Immunosorbent Assay (ELISA)

For assessment of the content of lipoteichoic acid (LTA) and endotoxin in fEV samples, commercially available ELISA kits were used (LTA‐ELISA kit, Abbexa, Leiden, the Netherlands and Pierce Endotoxin Quant Kit, Thermo Fisher Scientific, Waltham, USA) according to the manufacturer´s instructions and measured on a Varioskan Lux (Thermo Fisher Scientific, USA).

### 2.10 Western Blot

fEVs were lysed by adding 100 µL lysis buffer (1 M Tris/HCl pH 7.4, 1% Triton X‐100, 5 M NaCl, 1 mM PMSF, 4% Protease inhibitor cocktail [Roche Diagnostics, Mannheim, Germany]) to 2 × 10^9^ fEVs and snap‐freezing in liquid nitrogen. Protein lysates were centrifuged to remove debris, and samples were frozen at −80°C until further analysis. Protein concentration was determined by bicinchoninic acid assay (BCA assay, Thermo Fisher Scientific, Waltham, MA, USA) according to the manufacturer's instructions. Total proteins of 20 µg were separated by 10% SDS–PAGE and transferred to a polyvinylidenfluoride (PVDF) membrane (Merck Millipore, Burlington) with a wet blotting apparatus (40 V, 200 mA, and 150 W for about 16 h, BioRad, Feldkirchen, Germany). Membranes were blocked in 5% nonfat milk/PBS/0.1% Tween for 1 h and immune‐blotted for 24 h with the primary antibody (rabbit anti‐human β‐Actin 1:500 [clone D6A8, cat. No. 8457], Cell Signaling, Cambridge, USA) in 1% nonfat milk/PBS/0.1% Tween 20. After washing in TBST buffer, membranes were incubated for 1 h at room temperature with the secondary antibody (HRP‐linked anti‐rabbit IgG, Cell Signaling) in 1% nonfat milk/PBS/0.1% Tween 20 and then developed by a chemiluminescence reaction (Amersham chemiluminescence solution, GE Healthcare, Germany) using the iBright CL1000 Imaging System (Thermo Fisher Scientific).

### 2.11 DNA Extraction and qPCR

DNA was extracted from stool samples or fEV samples using the ZymoBIOMICS DNA Miniprep kit (ZymoResearch, Freiburg i. Br., Germany) according to manufacturer's instructions. DNA concentration was measured using a Varioskan LUX Microplate Reader (Thermo Fisher Scientific, Waltham, MA, USA) and adjusted to a concentration of 50 ng/µL for the qPCR.

The qPCR reaction was performed on a QuantStudio 3 PCR system (Thermo Fisher Scientific, Waltham, USA). All primers were purchased from MERCK (Darmstadt, Germany) with the following sequences: universal 16S forward AACTCAAAKGAATTGACGG and reverse CTCACRRCACGAGCTGAC; *Bacteroidetes* forward CRAACAGGATTAGATACCCT and reverse GGTAAGGTTCCTCGCGTAT; *Firmicutes* forward GGAGYATGTGGTTTAATTCGAAGCA and reverse AGCTGACGACAACCATGCAC; *Gammaproteobacteria* forward TCGTCAGCTCGTGTYGTGA and reverse CGTAAGGGCCATGATG; *Alphaproteobacteria* forward CIAGTGTAGAGGTGAAATT and reverse CCCCGTCAATTCCTTTGAGTT; *Actinobacteria* forward TACGGCCGCAAGGCTA and reverse TCRTCCCCACCTTCCTCCG; *Fusobacteria* forward AAGCGCGTCTAGGTGGTTATGT and reverse TGTAGTTCCGCTTACCTCTCCAG. The qPCR was performed using PowerUP SYBR Green Master Mix (Thermo Fisher Scientific, Waltham, USA). Annealing temperature of 61.5°C was used for all primer pairs. Melting curve analysis was used as quality control. Data collection was enabled at each increment of the melt curve. Analysis was performed using the QuantStudio Design & Analysis Software (Version 2.6.0; Thermo Fisher Scientific, Waltham, USA). The relative amount of each specific phylum was calculated in relation to the universal 16S load.

### 2.12 16s rRNA Sequencing and Bioinformatics

Miseq Illumina paired‐end sequencing reads were processed using a homemade custom pipeline. Sequencing reads were first decontaminated against the human genome using DeconSeq (v0.4.3) with the GRCh38/hg38 reference genome [27]. The processed reads were imported into QIIME 2 (v2023.5) [28]. Read quality was assessed using the generated interactive quality plots, which enabled the selection of trimming parameters for subsequent steps. Filtration of low‐quality reads and denoising steps were performed using the DADA2 plugin within QIIME 2 [29]. Duplicates were removed using derepFastq, and chimeric sequences were detected and removed with removeBimeraDenovo. Forward and reverse reads were merged using the mergePairs function to create amplicon sequence variants (ASVs) and sample‐by‐sequence observation matrix using makeSequenceTable. The taxonomic classification was performed using a Naive Bayesian classifier trained on the SILVA database version 138 [30] after aligning sequences using MAFFT and generating ASV phylogenetic trees using FastTree [31], which were built with the align‐to‐tree‐mafft‐fasttree plugin in QIIME 2. The generated QIIME 2 results, including feature tables, representative sequences, and taxonomic assignments, were exported into standard text outputs and BIOM tab‐separated file format [32] for downstream analyses.

All 16s data were analyzed in R (R Core Team, 2024) using PhyloSeq [33] and vegan packages. ASVs, which were not assigned to bacterial and archaeal kingdoms, were removed. In addition, any ASV detected in negative controls was strictly removed from all samples. Among 1278 ASVs, we only collected 5 ASVs in the negative controls sourced from surfaces, DNA extraction kits, and PCR. The ASV abundance in each sample was normalized using TSS (total sum scaling) method [34]. To assess the alpha diversity, we used the Shannon diversity index, which accounts for both diversity units’ richness and evenness. For beta diversity, the different microbiome groups were compared using the Bray–Curtis dissimilarity as ecological distance and the UniFrac phylogenetic distance weighted with the ASV abundance information. Permutational multivariate analysis of variance (PERMANOVA) was used to test for differences in beta diversity among groups. *p* values were adjusted for multiple comparisons using the Benjamini–Hochberg method. The differential abundance package DeSEq2 [35] was applied to identify important differentially expressed taxa from pairwise group comparisons, which subsequently were visualized in heatmaps.

### 2.13 Proteome Analyses

For proteome analyses, MACS‐isolated and fEV‐stimulated T cells were lysed by adding lysis buffer (Pierce RIPA buffer (Thermo Fisher Scientific), 1 mM PMSF, 1% protease inhibitor cocktail) on ice followed by snap‐freezing in liquid nitrogen. Each sample of 10 mg was digested in solution with trypsin as described in [36]. After desalting using C18 stage tips, extracted peptides were separated on an Easy‐nLC 1200 system coupled to a Q Exactive HFX mass spectrometer (Thermo Fisher Scientific) as described in [37] with slight modifications: The peptide mixtures were separated using a 90 min segmented gradient from 10% to 33% to 50% to 90% of HPLC solvent B (80% acetonitrile in 0.1% formic acid) in HPLC solvent A (0.1% formic acid) at a flow rate of 200 nL/min. The 12 most intense precursor ions were sequentially fragmented in each scan cycle using higher energy collisional dissociation (HCD) fragmentation. Acquired MS spectra were processed with MaxQuant software package version 2.2.0.0 with the integrated Andromeda search engine. Database search was performed against a target‐decoy Homo sapiens database obtained from Uniprot, containing 93.827 protein entries and 286 commonly observed contaminants. Peptide, protein, and modification site identifications were reported at a false discovery rate (FDR) of 0.01, estimated by the target/decoy approach. Data analysis was performed with the Perseus software version 2.0.11. Protein intensities were used for relative protein quantification.

### 2.14 Statistical Analysis

Statistical analysis was done using GraphPad Prism 9.4.1 (GraphPad Software, La Jolla, CA). Data were analyzed for Gaussian distribution using the Shapiro–Wilk test. Comparisons between two groups of unpaired and normally distributed data were analyzed using the unpaired *t*‐test; comparisons of two groups of unpaired and not‐normally distributed data were evaluated using the Mann–Whitney test. A *p* value <0.05 was considered as statistically significant.

## 3 Results

### 3.1 Isolation and Characterization of fEVs

In order to establish a simple protocol for isolation of fEVs from human feces, six slightly different protocols were tested with stool samples stored at −80°C (Table S1). After treatment of the samples according to the six protocols, the fEVs were isolated either by ultracentrifugation or by precipitation with ExoQuick. We found only slight but irrelevant differences in protein yield between the six protocols (Figure S1A). After purification of fEVs by ultracentrifugation, the protein yield was slightly higher for all protocols than after purification by precipitation (Figure S1A). Figure S1B shows size/concentration distribution, including the mode size of EVs isolated according to the six different protocols. Due to the simplest feasibility of protocol II in our setting, further experiments were carried out according to this protocol. In the next step, we investigated whether there were differences in protein yield between fresh stool, frozen stool, or stool stored in DNA/RNA shield. Again, there were only slight differences in the protein yield between these different storage methods. For fresh and frozen stool, protein yield was higher after isolation by ultracentrifugation than after isolation by precipitation. For stool stored in DNA/RNA shield, there were no differences between the two isolation methods (Figure S2A). No relevant differences were observed in fEV size and concentration between frozen stool and stool in DNA/RNA shield (Figure S2B,C) as assessed by NTA. Purification by precipitation resulted in a slightly higher yield of fEVs from both frozen stool and stool in DNA/RNA shield (Figure S2C). Figure S2D shows size/concentration distribution, including the mode size of EVs isolated from frozen stool and stool in DNA/RNA shield. On the basis of these results, we further used stool stored in DNA/RNA shield and purified fEVs according to protocol II and subsequent precipitation.

**FIGURE 1:**
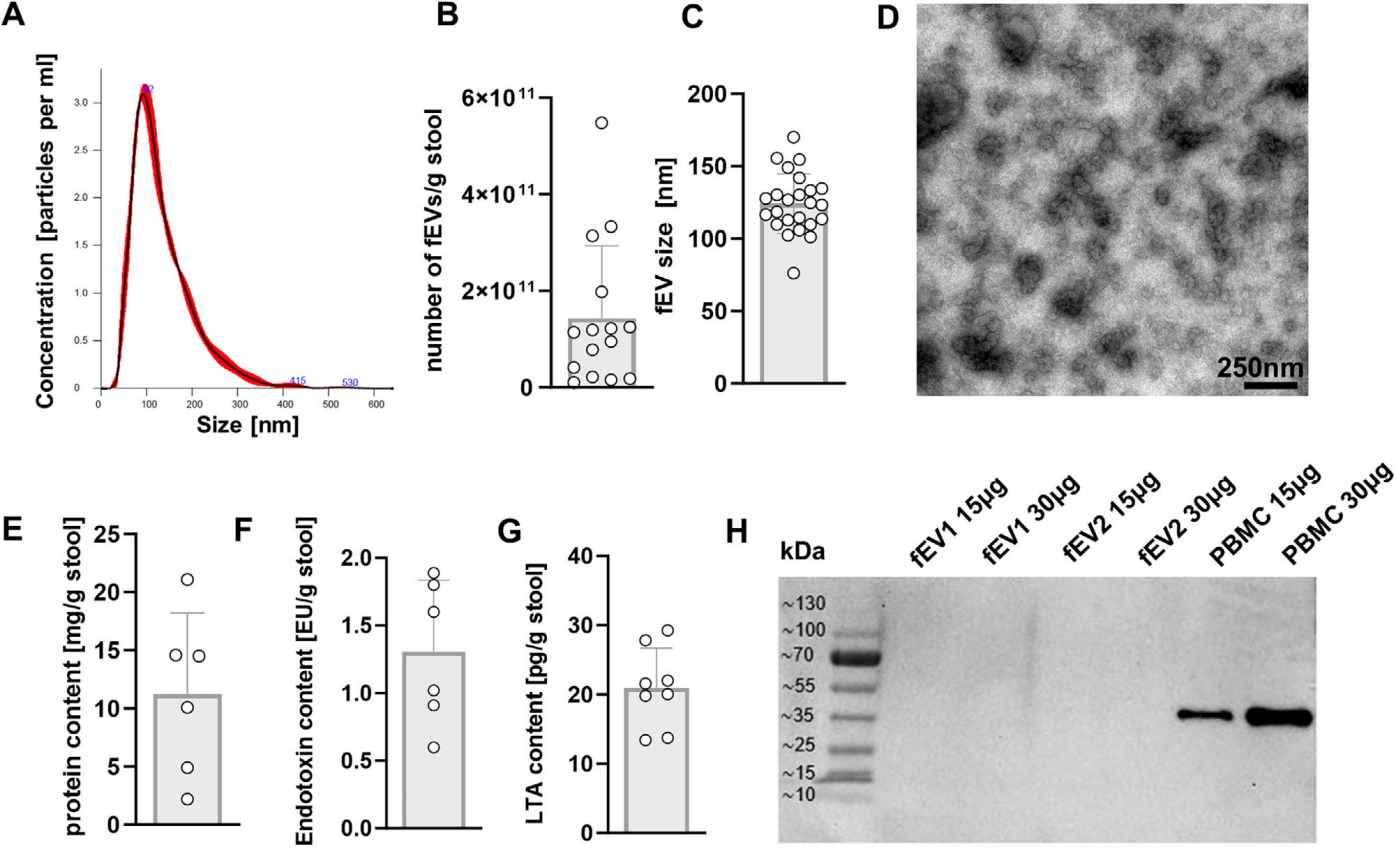
Characterization of fEVs isolated from stool samples. Fecal EVs were purified from stool samples stored in DNA/RNA Shield and characterized by NTA, TEM, BCA, ELISA, and Western blot. (A) Representative histogram and picture from NTA by NanoSight NS300. (B and C) Scatter plots with bars show (B) number of fEVs per gram stool and (C) fEV size in nm as assessed by NTA (*n* = 14). (D) Representative electron microscope image of fEVs isolated from stool. (E) Scatter plot with bars shows the protein content per gram of stool as assessed by BCA assay (*n* = 6). (F and G) Scatter diagrams with bars show endotoxin content (F) and LTA content (G) of fEVs as assessed by ELISA (*n* = 6–8). (H) Representative Western blot image shows expression of β‐Actin in two different fEV samples at two concentrations (15 and 30 µg) and in PBMC isolated from a healthy adult donor. LTA, lipoteichoic acid.

Purified fEVs were then characterized by NTA and TEM. NTA showed a concentration of median 1.1 × 10^11^ (range 1.1 × 10^10^ to 5.4 × 10^11^) fEVs per gram of stool and an average size of 124 nm (range 76–170 nm) (Figure 1A–C). Visualization of isolated fEVs by TEM confirmed the presence of typical round‐shaped vesicles within a size range of 26–121 nm (Figure 1D). The protein content of isolated fEVs assessed by BCA assay was a median of 2.0 mg/g stool (range 0.4–3.2 mg/g stool) (Figure 1E). Next, we analyzed the presence of Gram‐negative endotoxin and Gram‐positive LTA by ELISA. As expected, we found both in our isolated fEV samples (Figure 1F,G). We found no relevant expression of β‐actin in the fEV samples, which would indicate cellular contamination (Figure 1H). In proteome analyses of fEV samples, however, we did not only detect bacterial proteins but also some proteins of human origin including the EV markers CD9 and CD63, which means that the EVs we isolated are a mixture of bacterial EVs and host EVs. However, only 3% of the identified peptides could be mapped to the human proteome, suggesting that human EVs were present in our samples only to a minimal extent.

### 3.2 fEVs From Non‐Pregnant and Pregnant Women Differ Slightly in Their Bacterial Origin but Not Their Protein Content

**FIGURE 2:**
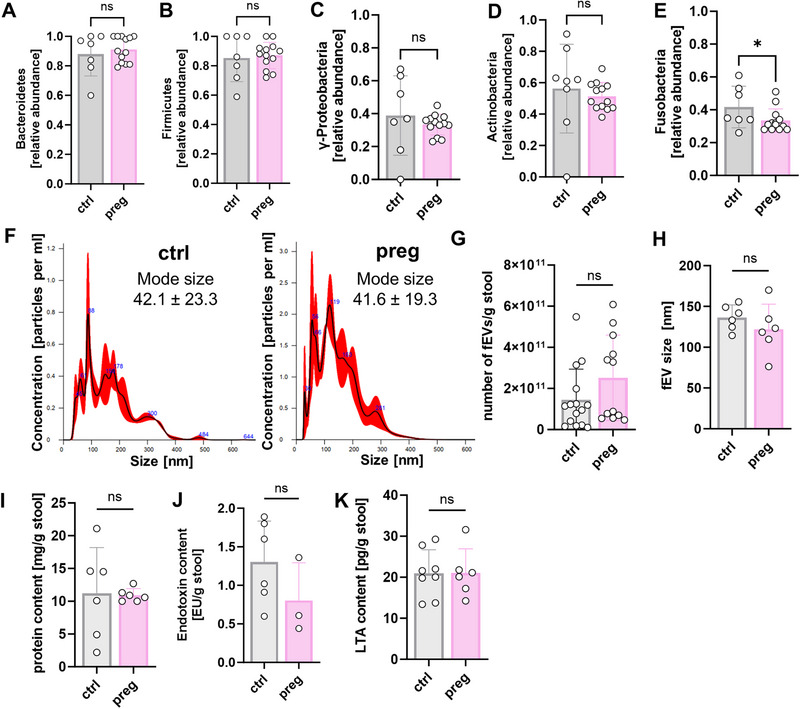
Bacterial composition and protein content of fEV samples from non‐pregnant controls and healthy pregnant women. (A–G) Fecal EVs were isolated from the stool of non‐pregnant controls (ctrl) and healthy pregnant women (preg) and analyzed by qPCR for the five main phyla of the microbiome. (A–E) Box plots showing the abundance of *Bacteroidetes* (A), *Firmicutes* (B), *γ‐Proteobacteria* (C), *Actinobacteria* (D), and *Fusobacteria* (E) relative to total 16s rRNA in fEVs from the stool of control subjects (grey box plots) and from the stool of pregnant women (rose box plots). *n* = 7–13, *p < 0.05, ns = not significant, Mann–Whitney test. (F–I) Fecal EVs were purified from stool samples from non‐pregnant controls (ctrl) and pregnant women (preg) stored in DNA/RNA Shield. Fecal EVs were analyzed by NTA, BCA, and ELISA. Scatter plots with bars show (F) the number of fEVs per gram of stool as assessed by NTA, (G) the protein content per gram of stool as assessed by BCA assay, and (H) endotoxin and (I) LTA content per gram of stool as assessed by ELISA. *n* = 3–15, ns = not significant, unpaired *t*‐test and Mann–Whitney test.

Next, we investigated whether fEVs isolated from the feces of pregnant women differed from fEVs isolated from the feces of non‐pregnant controls. We found slight differences in bacterial composition on a phylum level with decreased detection of *Fusobacteria*‐derived 16s rRNA in fEVs from pregnant women (relative abundance 0.42 ± 0.13 vs. 0.33 ± 0.07, *n* = 6–11, *p* < 0.05) as detected by qPCR (Figure 2A–E). 16s rRNA sequencing of individual samples showed significant differences in the abundance of a few taxa at a genus level. Although bacteria belonging to the genera *CAG:56*, *vadinBE97*, and *Tyzzerella* were found in increased amounts in the fEV samples of non‐pregnant women, bacteria belonging to the genera *Dorea*, *Izemoplasmatales*, *Eubacterium xylanophilum*, *CAG:352*, *Acidaminococcus*, and *Megasphera* were found in increased amounts in the fEV samples of pregnant women (Figure S3).

When looking at the protein content of fEVs isolated from feces of pregnant and non‐pregnant women, a similar total protein content was found in the EVs from feces of non‐pregnant controls and pregnant women (11.23 ± 6.96 mg/g for ctrl stool and 10.92 ± 1.01 mg/g for stool from pregnant women, *n* = 6–9, Figure 2G), a similar number of vesicles per gram of stool (median 1.14 × 10^11^ vs. 8.86 × 10^10^, *n* = 13–15, Figure 2F), and a similar size distribution (Figure 2G,H). Both endotoxin and LTA levels did also not differ between fEVs from non‐pregnant controls and pregnant women (1.30 ± 0.53 vs. 0.80 ± 0.049 EU/g stool for endotoxin and 20.96 ± 5.71 vs. 21.02 ± 5.89 pg/g stool for LTA, Figure 2H,I).

### 3.3 fEVs From Non‐Pregnant and Pregnant Women Differentially Modulate T Cell Phenotype

**FIGURE 3:**
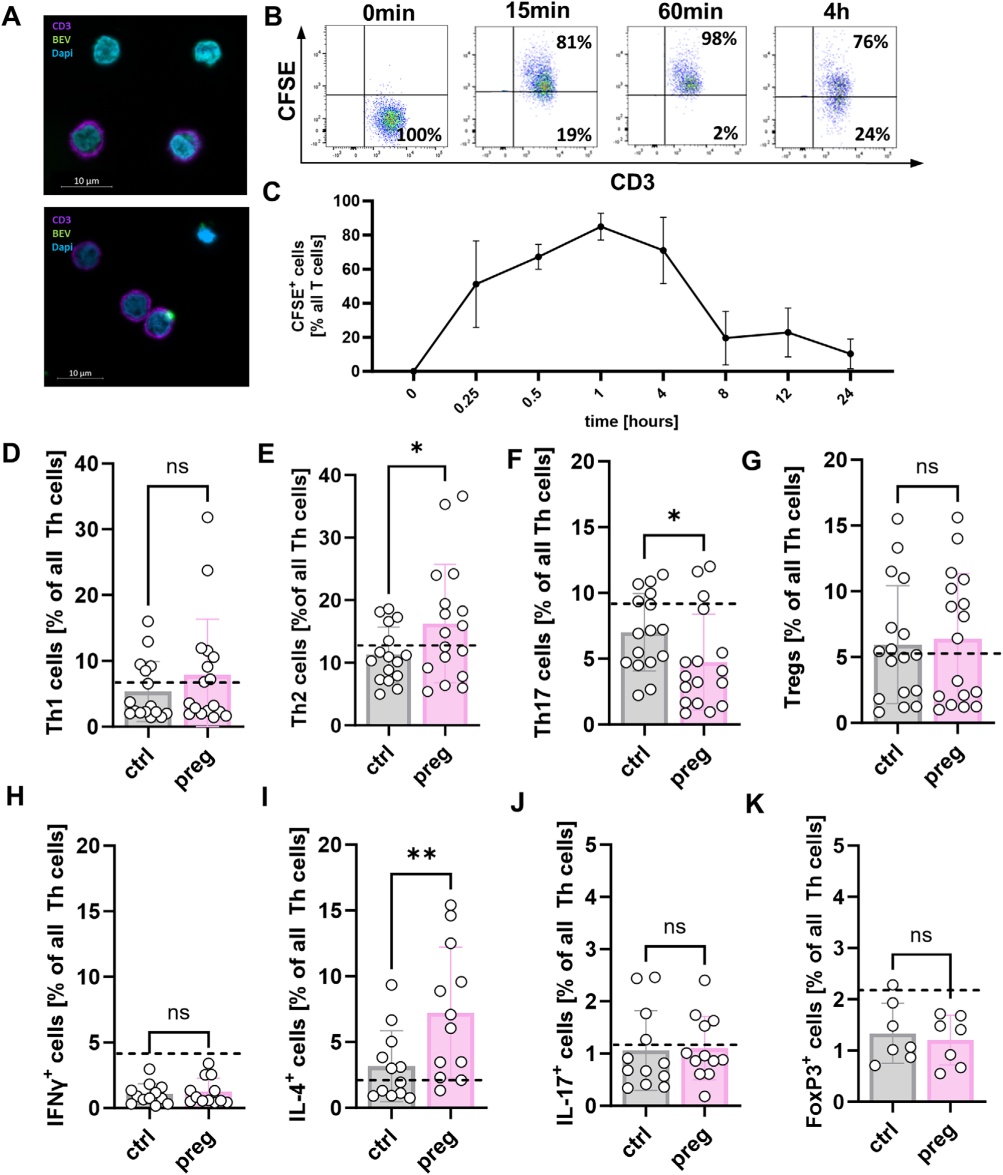
T cell phenotype after stimulation with fEVs. (A–C) Fecal EVs were stained with CFSE and added to freshly isolated PBMC. Uptake of fEV by T cells was analyzed by fluorescence microscopy and flow cytometry. (A) Representative fluorescence microscopy images of PBMC incubated with CFSE‐stained fEVs (green). T cells are immune‐stained with CD3 (violet). Nuclei are stained with DAPI (blue). Magnification 63×; scale bar 10 µm. (B) Representative pseudocolor plots of CD3 versus CFSE after 15 min, 60 min, and 4 h incubation of PBMC with CFSE‐stained fEVs showing CFSE‐positive T cells in the upper right quadrant. Cells were pre‐gated on living CD45^+^ cells. (C) Time diagram showing the percentage of CFSE‐positive T cells from all T cells after different times of incubation of PBMC with CFSE‐stained fEVs. (D–G) Fecal EVs were isolated from stool of non‐pregnant controls (ctrl, *n* = 11) and pregnant women (preg, *n* = 13) and added to freshly MACS‐isolated T cells from healthy adult donors (*n* = 5). T cells without fEV stimulation served as control. After 24 h of incubation, T cells were analyzed by flow cytometry. Scatter diagrams with bars show percentages of CCR4^−^/CXCR3^+^/CCR6^−^ T helper 1 cells (D), CCR4^+^/CXCR3^‐^/CCR6^−^ T helper 2 cells (E), CCR4^+^/CXCR3^‐^/CCR6^+^ T helper 17 cells (F), and CD25^+^ regulatory T cells (G) from all T helper cells in T cells stimulated with fEVs from non‐pregnant controls (gray bars) and from pregnant women (rose bars). Each symbol represents an individual fEV/T cell pair, and the mean is indicated. The proportion of corresponding T cell populations in unstimulated T cells is shown as a dashed line. *p < 0.05; ns = not significant. Mann–Whitney test and unpaired *t*‐test. (H–K) Fecal EVs were isolated from stool of non‐pregnant controls (ctrl, *n* = 13) and pregnant women (preg, *n* = 14) and added to freshly isolated PBMCs from healthy adult donors (*n* = 5). PBMC without fEV‐stimulation served as control. After 24 h of incubation, intracellular cytokines of T cells were analyzed by flow cytometry. Scatter diagrams with bars show percentages of IFNγ^+^ T cells (H), IL‐4^+^ T cells (I), IL‐17^+^ T cells (J), and Foxp3^+^ T cells (K) from all T helper cells in PBMC stimulated with fEV from non‐pregnant controls (gray bars) and from pregnant women (rose bars). Each symbol represents an individual fEV/PBMC pair, and the mean is indicated. The proportion of corresponding T cell populations in unstimulated T cells is shown as a dashed line. *p < 0.05; ns = not significant. Mann–Whitney test and unpaired *t*‐test. CFSE, carboxyfluorescein succinimidyl ester.

To investigate the immunomodulatory effects of fEVs, we established an *in vitro* culture model with fEVs and PBMC/T cells. For this purpose, we first stained fEVs with CFSE and then added them to freshly isolated PBMCs from healthy donors. In Figure 3A, representative fluorescence microscopy images of PBMC incubated with fEVs are shown. Figure S4 shows a negative staining control of PBMC without fEVs. CFSE‐stained fEVs were localized in the cytosol of immunostained T cells, demonstrating that fEVs are taken up by these cells. In time experiments, we showed by flow cytometry that fEV uptake by T cells began immediately after the addition of fEVs to PBMC (PBMC:fEVs = 1:1000) and reached a maximum after approx. 1 h. After 24 h hardly any fEVs were detectable in the T cells (Figure 3B,C). We then analyzed the proportions of the Th cell subpopulations Th1, Th2, Th17, and T_reg_ in MACS‐isolated T cells after 24 h of incubation with fEVs from non‐pregnant controls and from pregnant women (gating strategy shown in Figure S5). We found that the proportions of CCR4^−^/CXCR3^+^/CCR6^−^ Th1 cells and CD25^+^ T_regs_ in the culture were similar after incubation with fEVs from non‐pregnant and pregnant women (Figure 3D,G), whereas the proportion of CCR4^+^/CXCR3^‐^/CCR6^−^ Th2 cells was increased and that of CCR4^+^/CXCR3^‐^/CCR6^+^ Th17 cells decreased by fEVs from pregnant individuals compared to non‐pregnant controls (Figure 3E,F). Accordingly, the expression of the Th2 cytokine IL‐4 in T cells was upregulated after stimulation of PBMC with fEVs from pregnant women compared to stimulation with fEVs from non‐pregnant controls (Figure 3I), whereas there was no effect on IFN‐γ, IL‐17, and FoxP3 expression in Th cells (Figure 3H,J,K).

### 3.4 Quantitative Proteomics Reveal Differences in the T Cell Proteome after Stimulation With fEVs From Pregnant and Non‐Pregnant Women

**FIGURE 4:**
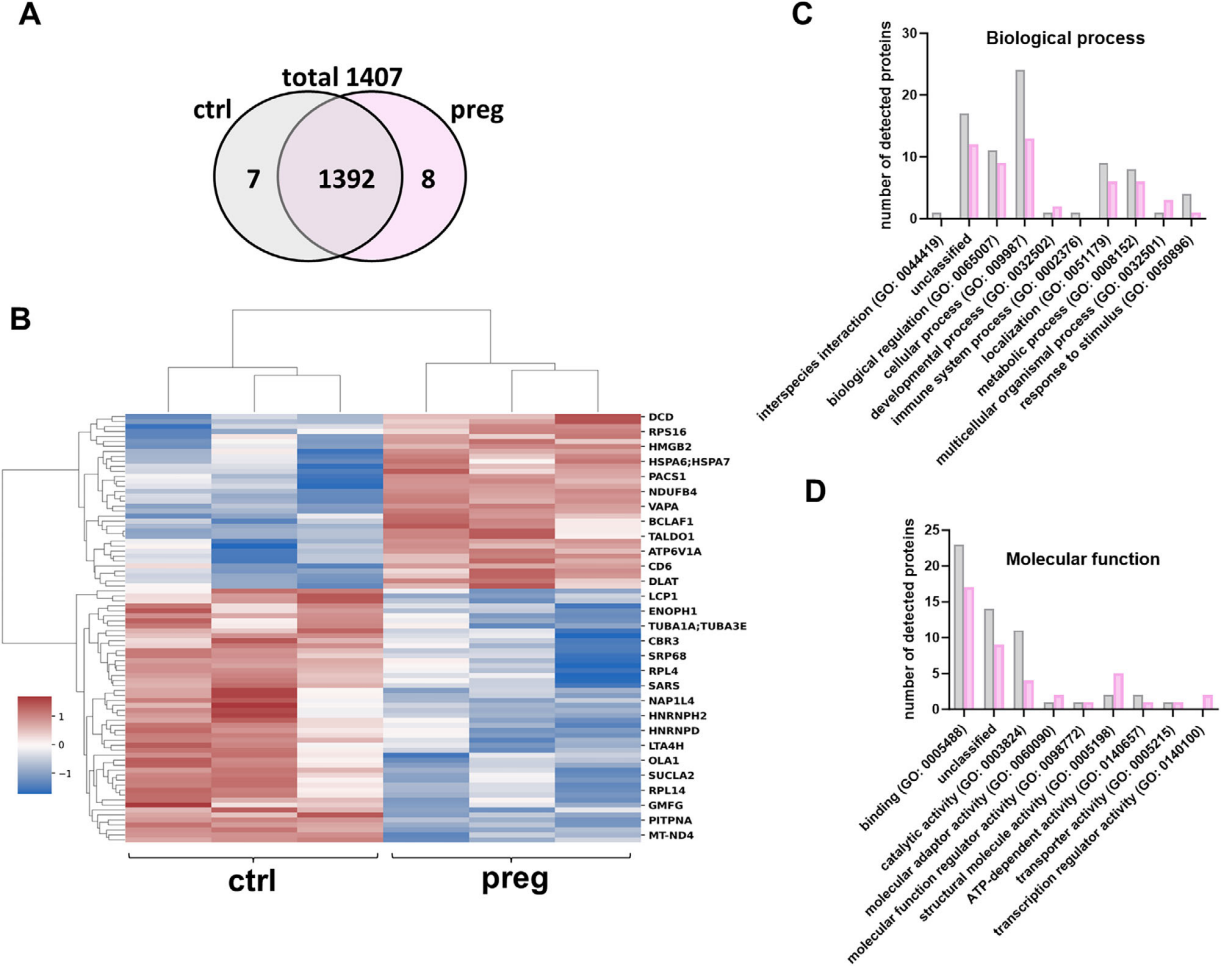
Proteome analyses of T cells stimulated with fEVs from non‐pregnant and pregnant women. Fecal EVs were isolated from stool of non‐pregnant controls (ctrl) and pregnant women (preg) and added to freshly MACS‐isolated T cells from healthy adult donors for 24 h and analyzed by mass spectrometry. (A) Venn diagram of shared and unique proteins identified in the proteome analyses of T cells stimulated with fEVs from non‐pregnant and pregnant women. (B) Heat map showing differentially expressed proteins in T cells stimulated with fEVs from non‐pregnant and pregnant women. (C and D) Bar graph showing the number of identified proteins in the proteome of T cells stimulated with fEVs from non‐pregnant (grey bars) and pregnant women (rose bars) distributed according to the biological process (C) and molecular functions by GO terms (D).

To uncover possible changes at protein level, we performed proteome analyses from isolated T cells after stimulation with fEVs from pregnant and non‐pregnant women. A total of 1407 proteins were detected, from which 7 were only detected in T cells stimulated with fEVs from non‐pregnant controls and 8 only in T cells stimulated with fEVs from pregnant women (Figure 4E). Expression of 51 proteins was significantly increased in T cells stimulated with fEVs from non‐pregnant controls, and expression of 35 proteins increased in T cells stimulated with fEVs from pregnant women (Figure 4A, Table S2). The GO molecular function and biological process categories of proteins that were upregulated in cells stimulated with fEVs from non‐pregnant and pregnant women are shown in Figure 4C,D. Consistent with our phenotypic results, T cells stimulated with fEVs from non‐pregnant women were found to upregulate proteins that may inhibit Th2 differentiation (Ras suppressor protein), induce Th1 and Th17 cells (Src kinase‐associated phosphoprotein 1, Interferon‐induced guanylate‐binding protein 2), and are associated with proinflammatory signaling pathways in T cells (Leukotriene A‐4 hydrolase, Plastin‐2, Crk‐like protein). Conversely, proteins that inhibit the differentiation of Th17 cells (calpastatin) and induce Th2 responses (Protein S100­A4) were upregulated in T cells stimulated with fEVs from pregnant women (highlighted in bold in Table S2).

## 4 Discussion

The aim of this study was to investigate *in vitro* whether and how fEVs influence immunological adaptation mechanisms during pregnancy. For this, we first established a protocol to isolate fEVs from preserved stool samples, as there are yet no uniform recommendations for the isolation of fEVs from stool samples [38]. Previous studies examining fEVs isolated them from fresh or immediately frozen stool samples [39, 40]. The method of isolating fEVs from stool samples preserved in DNA/RNA shield and stored at room temperature that we used here offers a logistical advantage for studies on fEVs, as samples can be collected by patients themselves and it is not necessary to maintain a cold chain. For the characterization of EVs, the International Society for Extracellular Vesicles (ISEV) recommends EM, the use of a single particle analyzer and protein quantification, and the detection of transmembrane and intracellular molecules, as well as the investigation of potential contaminants [38]. EM and NTA showed EVs with a size of 76–170 nm and a yield of approx. 1.1 × 10^11^ fEVs per gram of stool. Both are in the order of magnitude of results reported by others for fecal bacterial extracellular vesicles (BEVs) [41]. The detection of LTA and endotoxin by ELISA confirms the bacterial origin, and the lack of detection of relevant amounts of β‐actin by Western blot confirms a bacterial origin of the EVs isolated by our method. However, proteome analyses revealed that proteins of human origin, such as the human EV markers CD9 and CD63, were also present in the samples, albeit to a very little extent. With regard to the isolation method, studies with plasma show that the purity of EVs isolated by ultracentrifugation was poorer, but the yield of EVs was better compared to isolation by precipitation [42, 43]. This could not be confirmed in our experiments, as we observed similar yields after purification with both methods.

When comparing fEVs from the stool of non‐pregnant and pregnant women, we found differences at the phylum level with decreased *Fusobacteria* in the samples of pregnant women. Very little is known about how the microbiome changes from a non‐pregnant state to pregnancy. However, increased abundances of *Fusobacterium* were found to be associated with adverse pregnancy outcomes [44]. Recently, the bacterial composition of stool was shown to differ from the corresponding fEVs, so that it is not possible to draw direct conclusions from the bacterial composition of the fEVs on the bacterial composition of the microbiome. Larger studies are needed that focus on changes in the microbiome and its fEVs caused by pregnancy.

Our analyses showed a similar concentration of fEVs per gram of stool as well as similar size, total protein content, and content of LTA and endotoxin in fEV samples from pregnant women and non‐pregnant controls. An important limitation of our study must be mentioned at this point. We did not examine every single sample used in our experiments but only a random selection for the quantification of vesicle numbers, protein content, and endotoxin and LTA content. Furthermore, we had incomplete information about factors that may influence microbiome composition, such as diet, life style, or previous medication [45] from our study participants. Thus, a more detailed analysis of the content of the fEVs and a larger sample size with complete metadata would be desirable for further studies on their immunological effects during pregnancy.

We observed a very rapid uptake of fEVs by T cells with a maximum after only 60 min. Similar kinetics have been described, for example, for DCs [46]. Various mechanisms of fEV uptake have been described (reviewed in [47]). The pathway by which fEVs were taken up by T cells in our study was not investigated.

In our culture experiments with fEVs and T cells, we observed that fEVs from the stool of non‐pregnant and pregnant women had different effects on the phenotype of T cells. fEVs from pregnant women caused a shift in the Th cell profile away from Th17 cells and toward Th2 cells. It has been known for a long time that a shift of the Th cell profile toward Th2 occurs during healthy pregnancy and that the absence of this shift is associated with pregnancy complications such as miscarriage or PE [9]. Similar associations have been observed for Th17 cells [5, 48]. The altered Th cell profile with increased presence of Th2 cells and decreased presence of Th17 cells after stimulation of T cells with fEVs from pregnant women compared to fEVs from non‐pregnant women, which we demonstrated both by extracellular characterization of Th subpopulations and in a similar trend by intracellular cytokine staining, indicates favorable immunologic effects of fEVs from pregnant women on pregnancy. Corresponding to the phenotypic results, we found differential expression of several proteins involved in Th cell polarization/differentiation between T cells stimulated with fEVs from non‐pregnant controls and from pregnant women. The protein calpastatin, which was shown to suppress Th17 cells [49, 50], was found to be overexpressed in T cells after stimulation with fEVs from pregnant women, as was the protein S100A4, which has been described as altering the Th1/Th2 balance toward Th2 [51, 52]. Conversely, proteins associated with proinflammatory signaling pathways that shift the Th cell balance toward Th1 and Th17 were overexpressed in T cells stimulated with fEVs from non‐pregnant women. The two most upregulated proteins in samples with fEVs from non‐pregnant women were Crk‐like protein and Ras (Rat sarcoma) suppressor protein 1 (RSU1). Crk‐like protein forms complexes with the transcription factor signal transducer and activator of transcription 5 (STAT5), leading to translocation to the nucleus and inducing STAT5‐dependent gene transcription [53]. STAT5, in turn, together with Janus kinase 2 (JAK2), was reported to induce Th1 and Th17 responses [54]. The Ras suppressor protein 1 was initially found to suppress RAS‐dependent oncogenic transformation [55]. To our knowledge, little is known about the role of RSU1 in Th cell function. However, it has been shown that the RAS/ERK‐pathway positively regulates IL‐4 expression and Th2 differentiation [56, 57]. This means that overexpression of RSU1 could lead to any Th2 differentiation being blocked after stimulation with fEVs in non‐pregnant women. Beyond this, two other proteins overexpressed in T cells stimulated with fEVs from non‐pregnant individuals—Src kinase‐associated phosphoprotein 1 and interferon‐induced guanylate‐binding protein 2—have been implicated in Th17 differentiation [58, 59]. Although the exact mechanism of Th cell regulation by fEVs during pregnancy remains unclear, our data provide clues as to the signaling pathways through which the modulation may occur.

We have not identified the bacteria responsible for the different effects of fEVs from pregnant and non‐pregnant women on Th cell phenotype. However, others have shown for certain bacteria that they induce specific Th cell responses via EVs. For example, EVs from *E. coli* and *Staphylococcus aureus* induced proinflammatory Th1 and Th17 responses, whereas EVs from *Helicobacter pylori* elicited predominantly Th2 immune responses [60, 61, 62]. In 16s rRNA analyses, we found differences in the presence of 16s rRNA of certain bacteria between fEV samples from non‐pregnant and pregnant individuals. However, only a few samples were analyzed here, so that no general conclusions can be drawn about the bacteria responsible for the observed immunological effects. Which bacteria and their EVs play a particular role in the context of pregnancy immune regulation should be the subject of larger scale studies. Another limitation of our study is that fEVs also contained small amounts of host EVs, which means that the effects we observe could not necessarily be mediated solely by bacterial EVs. Hormonal changes, changes in the microbiome, or metabolic changes could cause intestinal host cells to release EVs with different functional properties during pregnancy than in non‐pregnant states. However, only about 3% of all peptides identified in our proteome analyses of fEVs were of human origin, making it highly unlikely that these significantly influence the effect on immune cells.

## 5 Conclusion

In summary, we show here for the first time that EVs isolated from the feces of pregnant women modulate T cells toward a regulatory phenotype favorable for pregnancy, indicating a direct interaction between the microbiome and the immune system during pregnancy. Further studies are needed to elucidate the underlying mechanisms in order to possibly develop microbiome‐targeted interventions for the prevention and treatment of immunological pregnancy complications.

## Author Contributions

All authors were involved in drafting the article or revising it critically for important intellectual content, and all authors approved the final version to be published. Stefanie Dietz‐Ziegler, Samantha Kewitz, Gabriele Kaiser, Jessica Rühle, Alexander Marmé, Alexander Dalpke, Bachar Cheaib, Jan Pauluschke‐Fröhlich, Melanie Henes, Ana Velic, Andreas Pich, Anneli Vollert, Martin Schaller, Felix Knab, Trim Lajqi, Christian F. Poets and Natascha Köstlin‐Gille contributed to the acquisition of data, analysis, and interpretation of data. Christian Gille and Christian F. Poets provided critical feedback on intellectual content. Natascha Köstlin‐Gille conceived the study and wrote the article.

## Conflicts of Interest

The authors declare no conflicts of interest.

## Peer Review

The peer review history for this article is available at https://publons.com/publon/10.1002/eji.70056.

## Supporting information




**Supporting file 1**: eji70056‐sup‐0001‐Figures.pdf;

**Supporting file 2**: eji70056‐sup‐0002‐Tables.docx.

## Data Availability

The datasets generated during and/or analyzed during the current study are available from the corresponding author upon reasonable requests.

## References

[1] R. L. Goldenberg , J. F. Culhane , J. D. Iams , and R. Romero , “Epidemiology and Causes of Preterm Birth,” Lancet 371, no. 9606 (2008): 75–84. 18177778 10.1016/S0140-6736(08)60074-4 PMC7134569

[2] C. W. Redman and I. L. Sargent , “Immunology of Pre‐Eclampsia,” American Journal of Reproductive Immunology 63, no. 6 (2010): 534–543. 20331588 10.1111/j.1600-0897.2010.00831.x

[3] L. Liu , H. L. Johnson , S. Cousens , et al., “Global, Regional, and National Causes of Child Mortality: An Updated Systematic Analysis for 2010 With Time Trends Since 2000,” Lancet 379, no. 9832 (2012): 2151–2161. 22579125 10.1016/S0140-6736(12)60560-1

[4] M. Kuhnert , R. Strohmeier , M. Stegmuller , and E. Halberstadt , “Changes in Lymphocyte Subsets During Normal Pregnancy,” European Journal of Obstetrics, Gynecology, and Reproductive Biology 76, no. 2 (1998): 147–151. 9481564 10.1016/s0301-2115(97)00180-2

[5] S. Saito , A. Nakashima , T. Shima , and M. Ito , “Th1/Th2/Th17 and Regulatory T‐Cell Paradigm in Pregnancy,” American Journal of Reproductive Immunology 63, no. 6 (2010): 601–610. 20455873 10.1111/j.1600-0897.2010.00852.x

[6] M. P. Piccinni , M. G. Giudizi , R. Biagiotti , et al., “Progesterone Favors the Development of Human T Helper Cells Producing Th2‐Type Cytokines and Promotes Both IL‐4 Production and Membrane CD30 Expression in Established Th1 Cell Clones,” Journal of Immunology 155, no. 1 (1995): 128–133. 7541410

[7] D. A. Somerset , Y. Zheng , M. D. Kilby , D. M. Sansom , and M. T. Drayson , “Normal Human Pregnancy Is Associated With an Elevation in the Immune Suppressive CD25+ CD4+ Regulatory T‐Cell Subset,” Immunology 112, no. 1 (2004): 38–43. 15096182 10.1111/j.1365-2567.2004.01869.x PMC1782465

[8] V. R. Aluvihare , M. Kallikourdis , and A. G. Betz , “Regulatory T Cells Mediate Maternal Tolerance to the Fetus,” Nature Immunology 5, no. 3 (2004): 266–271. 14758358 10.1038/ni1037

[9] R. Raghupathy , M. Makhseed , F. Azizieh , N. Hassan , M. Al‐Azemi , and E. Al‐Shamali , “Maternal Th1‐ and Th2‐type Reactivity to Placental Antigens in Normal Human Pregnancy and Unexplained Recurrent Spontaneous Abortions,” Cellular Immunology 196, no. 2 (1999): 122–130. 10527564 10.1006/cimm.1999.1532

[10] M. Frascoli , L. Coniglio , R. Witt , et al., “Alloreactive Fetal T Cells Promote Uterine Contractility in Preterm Labor via IFN‐Gamma and TNF‐Alpha,” Science Translational Medicine 10, no. 438 (2018): eaan2263. 29695455 10.1126/scitranslmed.aan2263 PMC6449052

[11] G. Toldi , J. Rigo Jr. , B. Stenczer , B. Vasarhelyi , and A. Molvarec , “Increased Prevalence of IL‐17‐Producing Peripheral Blood Lymphocytes in Pre‐Eclampsia,” American Journal of Reproductive Immunology 66, no. 3 (2011): 223–229. 21306467 10.1111/j.1600-0897.2011.00987.x

[12] G. Toldi , P. Svec , B. Vasarhelyi , et al., “Decreased Number of FoxP3+ Regulatory T Cells in Preeclampsia,” Acta Obstetricia Et Gynecologica Scandinavica 87, no. 11 (2008): 1229–1233. 19016357 10.1080/00016340802389470

[13] D. C. Cornelius , L. M. Amaral , A. Harmon , et al., “An Increased Population of Regulatory T Cells Improves the Pathophysiology of Placental Ischemia in a Rat Model of Preeclampsia,” American Journal of Physiology Regulatory, Integrative and Comparative Physiology 309, no. 8 (2015): R884–R891. 26290102 10.1152/ajpregu.00154.2015 PMC4666948

[14] D. C. Cornelius , L. M. Amaral , K. Wallace , et al., “Reduced Uterine Perfusion Pressure T‐Helper 17 Cells Cause Pathophysiology Associated With Preeclampsia During Pregnancy,” American Journal of Physiology Regulatory, Integrative and Comparative Physiology 311, no. 6 (2016): R1192–R1199. 27784685 10.1152/ajpregu.00117.2016 PMC5256975

[15] S. J. Ott , M. Musfeldt , D. F. Wenderoth , et al., “Reduction in Diversity of the Colonic Mucosa Associated Bacterial Microflora in Patients With Active Inflammatory Bowel Disease,” Gut 53, no. 5 (2004): 685–693. 15082587 10.1136/gut.2003.025403 PMC1774050

[16] V. Pascal , M. Pozuelo , N. Borruel , et al., “A Microbial Signature for Crohn's Disease,” Gut 66, no. 5 (2017): 813–822. 28179361 10.1136/gutjnl-2016-313235 PMC5531220

[17] I. A. Harsch and P. C. Konturek , “The Role of Gut Microbiota in Obesity and Type 2 and Type 1 Diabetes Mellitus: New Insights Into “Old” Diseases,” Medical Sciences (Basel) 6, no. 2 (2018): 32. 10.3390/medsci6020032 PMC6024804 29673211

[18] A. Pascale , N. Marchesi , S. Govoni , A. Coppola , and C. Gazzaruso , “The Role of Gut Microbiota in Obesity, Diabetes Mellitus, and Effect of Metformin: New Insights Into Old Diseases,” Current Opinion in Pharmacology 49 (2019): 1–5. 31015106 10.1016/j.coph.2019.03.011

[19] A. N. Thorburn , C. I. McKenzie , S. Shen , et al., “Evidence That Asthma Is a Developmental Origin Disease Influenced by Maternal Diet and Bacterial Metabolites,” Nature Communications 6 (2015): 7320. 10.1038/ncomms8320 26102221

[20] O. Koren , J. K. Goodrich , T. C. Cullender , et al., “Host Remodeling of the Gut Microbiome and Metabolic Changes During Pregnancy,” Cell 150, no. 3 (2012): 470–480. 22863002 10.1016/j.cell.2012.07.008 PMC3505857

[21] N. Diaz‐Garrido , J. Badia , and L. Baldoma , “Microbiota‐Derived Extracellular Vesicles in Interkingdom Communication in the Gut,” Journal of Extracellular Vesicles 10, no. 13 (2021): e12161. 34738337 10.1002/jev2.12161 PMC8568775

[22] S. K. Mazmanian , C. H. Liu , A. O. Tzianabos , and D. L. Kasper , “An Immunomodulatory Molecule of Symbiotic Bacteria Directs Maturation of the Host Immune System,” Cell 122, no. 1 (2005): 107–118. 16009137 10.1016/j.cell.2005.05.007

[23] Y. Shen , M. L. Giardino Torchia , G. W. Lawson , C. L. Karp , J. D. Ashwell , and S. K. Mazmanian , “Outer Membrane Vesicles of a Human Commensal Mediate Immune Regulation and Disease Protection,” Cell Host & Microbe 12, no. 4 (2012): 509–520. 22999859 10.1016/j.chom.2012.08.004 PMC3895402

[24] S. H. Yoon , S. O. Yun , J. Y. Park , et al., “Selective Addition of CXCR3(+) CCR4(−) CD4(+) Th1 Cells Enhances Generation of Cytotoxic T Cells by Dendritic Cells In Vitro,” Experimental & Molecular Medicine 41, no. 3 (2009): 161–170. 19293635 10.3858/emm.2009.41.3.019 PMC2679244

[25] J. Mjosberg , G. Berg , M. C. Jenmalm , and J. Ernerudh , “FOXP3+ Regulatory T Cells and T Helper 1, T Helper 2, and T Helper 17 Cells in Human Early Pregnancy Decidua,” Biology of Reproduction 82, no. 4 (2010): 698–705. 20018909 10.1095/biolreprod.109.081208

[26] S. Clark , E. Page , T. Ford , et al., “Reduced T(H)1/T(H)17 CD4 T‐Cell Numbers Are Associated With Impaired Purified Protein Derivative‐Specific Cytokine Responses in Patients With HIV‐1 Infection,” Journal of Allergy and Clinical Immunology 128, no. 4 (2011): 838–846.e5. 21745684 10.1016/j.jaci.2011.05.025

[27] R. Schmieder and R. Edwards , “Fast Identification and Removal of Sequence Contamination From Genomic and Metagenomic Datasets,” PLoS ONE 6, no. 3 (2011): e17288. 21408061 10.1371/journal.pone.0017288 PMC3052304

[28] E. Bolyen , J. R. Rideout , M. R. Dillon , et al., “Reproducible, Interactive, Scalable and Extensible Microbiome Data Science Using QIIME 2,” Nature Biotechnology 37, no. 8 (2019): 852–857. 10.1038/s41587-019-0209-9 PMC7015180 31341288

[29] B. J. Callahan , P. J. McMurdie , M. J. Rosen , A. W. Han , A. J. Johnson , and S. P. Holmes , “DADA2: High‐Resolution Sample Inference From Illumina Amplicon Data,” Nature Methods 13, no. 7 (2016): 581–583. 27214047 10.1038/nmeth.3869 PMC4927377

[30] C. Quast , E. Pruesse , P. Yilmaz , et al., “The SILVA Ribosomal RNA Gene Database Project: Improved Data Processing and Web‐Based Tools,” Nucleic Acids Research 41, no. Database issue (2013): D590–D596. 23193283 10.1093/nar/gks1219 PMC3531112

[31] M. N. Price , P. S. Dehal , and A. P. Arkin , “FastTree 2‐Approximately Maximum‐Likelihood Trees for Large Alignments,” PLoS ONE 5, no. 3 (2010): e9490. 20224823 10.1371/journal.pone.0009490 PMC2835736

[32] D. McDonald , J. C. Clemente , J. Kuczynski , et al., “The Biological Observation Matrix (BIOM) Format or: How I Learned to Stop Worrying and Love the Ome‐Ome,” Gigascience 1, no. 1 (2012): 7. 23587224 10.1186/2047-217X-1-7 PMC3626512

[33] P. J. McMurdie and S. Holmes , “Phyloseq: An R Package for Reproducible Interactive Analysis and Graphics of Microbiome Census Data,” PLoS ONE 8, no. 4 (2013): e61217. 23630581 10.1371/journal.pone.0061217 PMC3632530

[34] M. A. Dillies , A. Rau , J. Aubert , et al., “A Comprehensive Evaluation of Normalization Methods for Illumina High‐Throughput RNA Sequencing Data Analysis,” Briefings in Bioinformatics 14, no. 6 (2013): 671–683. 22988256 10.1093/bib/bbs046

[35] M. I. Love , W. Huber , and S. Anders , “Moderated Estimation of Fold Change and Dispersion for RNA‐Seq Data With DESeq2,” Genome Biology 15, no. 12 (2014): 550. 25516281 10.1186/s13059-014-0550-8 PMC4302049

[36] N. Borchert , C. Dieterich , K. Krug , et al., “Proteogenomics of *Pristionchus pacificus* Reveals Distinct Proteome Structure of Nematode Models,” Genome Research 20, no. 6 (2010): 837–846. 20237107 10.1101/gr.103119.109 PMC2877580

[37] K. Kliza , C. Taumer , I. Pinzuti , et al., “Internally Tagged Ubiquitin: A Tool to Identify Linear Polyubiquitin‐Modified Proteins by Mass Spectrometry,” Nature Methods 14, no. 5 (2017): 504–512. 28319114 10.1038/nmeth.4228

[38] C. Thery , K. W. Witwer , E. Aikawa , et al., “Minimal Information for Studies of Extracellular Vesicles 2018 (MISEV2018): A Position Statement of the International Society for Extracellular Vesicles and Update of the MISEV2014 Guidelines,” Journal of Extracellular Vesicles 7, no. 1 (2018): 1535750. 30637094 10.1080/20013078.2018.1535750 PMC6322352

[39] L. Fizanne , A. Villard , N. Benabbou , et al., “Faeces‐Derived Extracellular Vesicles Participate in the Onset of Barrier Dysfunction Leading to Liver Diseases,” Journal of Extracellular Vesicles 12, no. 2 (2023): e12303. 36708245 10.1002/jev2.12303 PMC9883837

[40] C. C. Li , W. F. Hsu , P. C. Chiang , M. C. Kuo , A. M. Wo , and Y. J. Tseng , “Characterization of Markers, Functional Properties, and Microbiome Composition in Human Gut‐Derived Bacterial Extracellular Vesicles,” Gut Microbes 15, no. 2 (2023): 2288200. 38038385 10.1080/19490976.2023.2288200 PMC10730231

[41] J. Tulkens , O. De Wever , and A. Hendrix , “Analyzing Bacterial Extracellular Vesicles in Human Body Fluids by Orthogonal Biophysical Separation and Biochemical Characterization,” Nature Protocols 15, no. 1 (2020): 40–67. 31776460 10.1038/s41596-019-0236-5

[42] J. Stam , S. Bartel , R. Bischoff , and J. C. Wolters , “Isolation of Extracellular Vesicles With Combined Enrichment Methods,” Journal of Chromatography B, Analytical Technologies in the Biomedical and Life Sciences 1169 (2021): 122604. 33713953 10.1016/j.jchromb.2021.122604

[43] Y. Tian , M. Gong , Y. Hu , et al., “Quality and Efficiency Assessment of Six Extracellular Vesicle Isolation Methods by Nano‐Flow Cytometry,” Journal of Extracellular Vesicles 9, no. 1 (2020): 1697028. 31839906 10.1080/20013078.2019.1697028 PMC6896440

[44] X. Chen , P. Li , M. Liu , et al., “Gut Dysbiosis Induces the Development of Pre‐Eclampsia Through Bacterial Translocation,” Gut 69, no. 3 (2020): 513–522. 31900289 10.1136/gutjnl-2019-319101

[45] M. Shabani , A. Ghoshehy , A. M. Mottaghi , et al., “The Relationship Between Gut Microbiome and Human Diseases: Mechanisms, Predisposing Factors and Potential Intervention,” Frontiers in Cellular and Infection Microbiology 15 (2025): 1516010. 40395507 10.3389/fcimb.2025.1516010 PMC12089137

[46] M. Mehanny , M. Koch , C. M. Lehr , and G. Fuhrmann , “Streptococcal Extracellular Membrane Vesicles Are Rapidly Internalized by Immune Cells and Alter Their Cytokine Release,” Frontiers in Immunology 11 (2020): 80. 32117243 10.3389/fimmu.2020.00080 PMC7034238

[47] E. J. O'Donoghue and A. M. Krachler , “Mechanisms of Outer Membrane Vesicle Entry Into Host Cells,” Cellular Microbiology 18, no. 11 (2016): 1508–1517. 27529760 10.1111/cmi.12655 PMC5091637

[48] S. K. Lee , J. Y. Kim , M. Lee , A. Gilman‐Sachs , and J. Kwak‐Kim , “Th17 and Regulatory T Cells in Women With Recurrent Pregnancy Loss,” American Journal of Reproductive Immunology 67, no. 4 (2012): 311–318. 22380579 10.1111/j.1600-0897.2012.01116.x

[49] M. Iguchi‐Hashimoto , T. Usui , H. Yoshifuji , et al., “Overexpression of a Minimal Domain of Calpastatin Suppresses IL‐6 Production and Th17 Development via Reduced NF‐kappaB and Increased STAT5 Signals,” PLoS ONE 6, no. 10 (2011): e27020. 22046434 10.1371/journal.pone.0027020 PMC3203168

[50] E. Letavernier , B. Dansou , M. Lochner , et al., “Critical Role of the Calpain/Calpastatin Balance in Acute Allograft Rejection,” European Journal of Immunology 41, no. 2 (2011): 473–484. 21268016 10.1002/eji.201040437

[51] B. Grum‐Schwensen , J. Klingelhofer , M. Beck , et al., “S100A4‐Neutralizing Antibody Suppresses Spontaneous Tumor Progression, Pre‐Metastatic Niche Formation and Alters T‐Cell Polarization Balance,” BMC cancer 15 (2015): 44. 25884510 10.1186/s12885-015-1034-2 PMC4335362

[52] S. Bruhn , Y. Fang , F. Barrenas , et al., “A Generally Applicable Translational Strategy Identifies S100A4 as a Candidate Gene in Allergy,” Science Translational Medicine 6, no. 218 (2014): 218ra4. 10.1126/scitranslmed.3007410 PMC4539009 24401939

[53] E. N. Fish , S. Uddin , M. Korkmaz , B. Majchrzak , B. J. Druker , and L. C. Platanias , “Activation of a CrkL‐STAT5 Signaling Complex by Type I Interferons,” Journal of Biological Chemistry 274, no. 2 (1999): 571–573. 9872990 10.1074/jbc.274.2.571

[54] Y. Wei , Z. Braunstein , J. Chen , et al., “JAK2/STAT5 Inhibition Protects Mice From Experimental Autoimmune Encephalomyelitis by Modulating T Cell Polarization,” International Immunopharmacology 120 (2023): 110382. 37269741 10.1016/j.intimp.2023.110382

[55] M. L. Cutler , R. H. Bassin , L. Zanoni , and N. Talbot , “Isolation of Rsp‐1, a Novel cDNA Capable of Suppressing v‐Ras Transformation,” Molecular and Cellular Biology 12, no. 9 (1992): 3750–3756. 1508180 10.1128/mcb.12.9.3750-3756.1992 PMC360236

[56] M. Yamashita , R. Shinnakasu , H. Asou , et al., “Ras‐ERK MAPK Cascade Regulates GATA3 Stability and Th2 Differentiation Through Ubiquitin‐Proteasome Pathway,” Journal of Biological Chemistry 280, no. 33 (2005): 29409–29419. 15975924 10.1074/jbc.M502333200

[57] E. Y. So , J. Oh , J. Y. Jang , J. H. Kim , and C. E. Lee , “Ras/Erk Pathway Positively Regulates Jak1/STAT6 Activity and IL‐4 Gene Expression in Jurkat T Cells,” Molecular Immunology 44, no. 13 (2007): 3416–3426. 17433443 10.1016/j.molimm.2007.02.022

[58] D. I. Kotov , J. S. Mitchell , T. Pengo , et al., “TCR Affinity Biases Th Cell Differentiation by Regulating CD25, Eef1e1, and Gbp2,” Journal of Immunology 202, no. 9 (2019): 2535–2545. 10.4049/jimmunol.1801609 PMC6478541 30858199

[59] X. Smith , A. Taylor , and C. E. Rudd , “T‐Cell Immune Adaptor SKAP1 Regulates the Induction of Collagen‐Induced Arthritis in Mice,” Immunology Letters 176 (2016): 122–127. 27181093 10.1016/j.imlet.2016.04.007 PMC4965781

[60] M. R. Kim , S. W. Hong , E. B. Choi , et al., “ *Staphylococcus aureus*‐Derived Extracellular Vesicles Induce Neutrophilic Pulmonary Inflammation via Both Th1 and Th17 Cell Responses,” Allergy 67, no. 10 (2012): 1271–1281. 22913540 10.1111/all.12001

[61] O. Y. Kim , B. S. Hong , K. S. Park , et al., “Immunization With *Escherichia coli* Outer Membrane Vesicles Protects Bacteria‐Induced Lethality via Th1 and Th17 Cell Responses,” Journal of Immunology 190, no. 8 (2013): 4092–4102. 10.4049/jimmunol.1200742 23514742

[62] Q. Liu , X. Li , Y. Zhang , et al., “Orally‐Administered Outer‐Membrane Vesicles From *Helicobacter pylori* Reduce *H. pylori* Infection Via Th2‐Biased Immune Responses in Mice,” Pathogens and Disease 77, no. 5 (2019): ftz050. 31504509 10.1093/femspd/ftz050

[63] T. Lajqi , N. Kostlin‐Gille , S. Hillmer , et al., “Gut Microbiota‐Derived Small Extracellular Vesicles Endorse Memory‐Like Inflammatory Responses in Murine Neutrophils,” Biomedicines 10, no. 2 (2022): 442. 35203650 10.3390/biomedicines10020442 PMC8962420

[64] J. Y. Park , C. S. Kang , H. C. Seo , et al., “Bacteria‐Derived Extracellular Vesicles in Urine as a Novel Biomarker for Gastric Cancer: Integration of Liquid Biopsy and Metagenome Analysis,” Cancers (Basel) 13, no. 18 (2021): 4687. 34572913 10.3390/cancers13184687 PMC8468964

[65] X. J. Gao , T. Li , B. Wei , et al., “Bacterial Outer Membrane Vesicles From Dextran Sulfate Sodium‐Induced Colitis Differentially Regulate Intestinal UDP‐Glucuronosyltransferase 1A1 Partially Through Toll‐Like Receptor 4/Mitogen‐Activated Protein Kinase/Phosphatidylinositol 3‐Kinase Pathway,” Drug Metabolism and Disposition: The Biological Fate of Chemicals 46, no. 3 (2018): 292–302. 29311138 10.1124/dmd.117.079046

[66] E. Alpdundar Bulut , B. Bayyurt Kocabas , V. Yazar , et al., “Human Gut Commensal Membrane Vesicles Modulate Inflammation by Generating M2‐Like Macrophages and Myeloid‐Derived Suppressor Cells,” Journal of Immunology 205, no. 10 (2020): 2707–2718. 10.4049/jimmunol.2000731 33028617

[67] C. S. Kang , M. Ban , E. J. Choi , et al., “Extracellular Vesicles Derived From Gut Microbiota, Especially *Akkermansia muciniphila*, Protect the Progression of Dextran Sulfate Sodium‐Induced Colitis,” PLoS ONE 8, no. 10 (2013): e76520. 24204633 10.1371/journal.pone.0076520 PMC3811976

