# Supplementary material for: Extracellular Vesicles Derived From the Feces of Pregnant Women Modulate T Cells Toward a Pregnancy‐Supportive Phenotype In Vitro
Source: Eur J Immunol. 2025 Sep 19;55(9):e70056. doi: 10.1002/eji.70056 (PMC12447864; doi:10.1002/eji.70056)
Supplement: Supplementary file 1 — Supporting file 1: eji70056‐sup‐0001‐Figures.pdf; [file EJI-55-e70056-s001.pdf]

Supplementary Figure 1

A

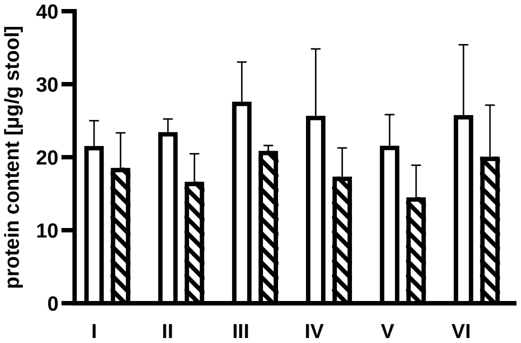

B

I	II	III	IV	V	VI
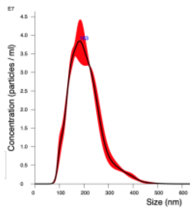	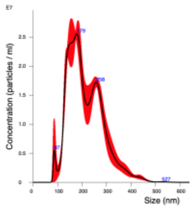	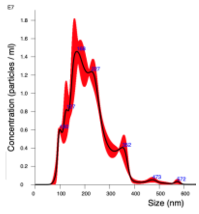	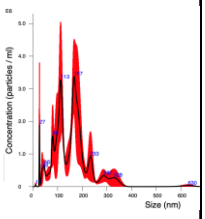	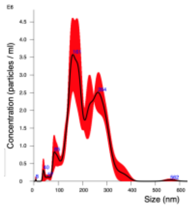	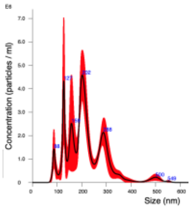
Mode sizes:					
182.2 nm	178.8 nm	167.5 nm	166.8 nm	160.2 nm	202.0 nm

Supplementary Figure 1: Comparison of different protocols and storage methods for fEV isolation from human stool samples

(A) Fecal EVs were purified from stool samples frozen at -80°C according to six different protocols. In the last step of each protocol either ultracentrifugation or precipitation was performed. Bar graph shows protein content of fEVs per gram stool after application of the six protocols and then either ultracentrifugation (empty bars) or precipitation (striped bars). (B) Representative histograms from NTA by NanoSight NS300.

Supplementary Figure 2

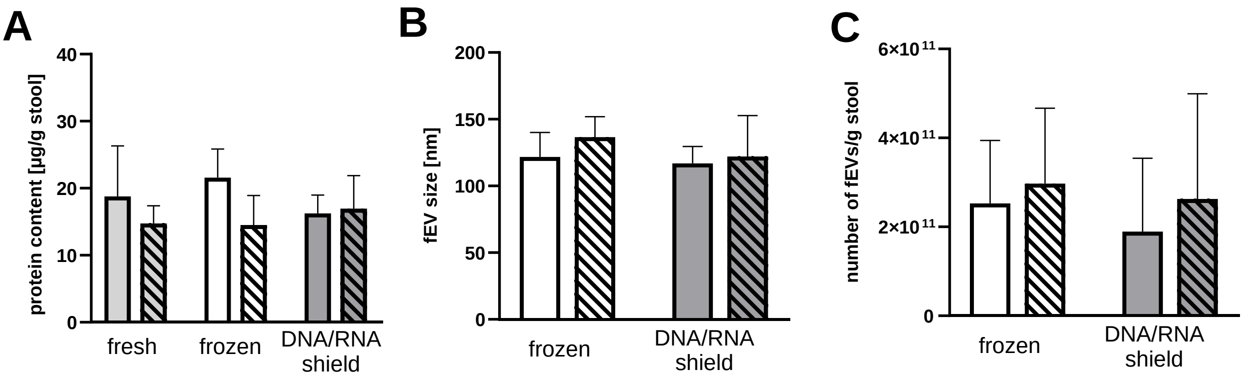

D

II	Fresh stool	Frozen stool	Preserved stool
Precipitation (ExoQuick)	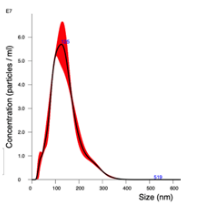	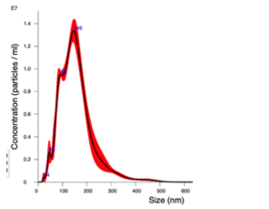	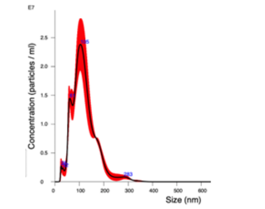
Mode	65.9 ± 36.6 nm (n=3)	105.8 ± 45.3 nm (n=7)	70.8 ± 29.2 nm (n=6)
Ultracentrifugation	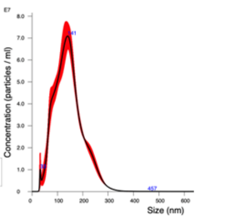	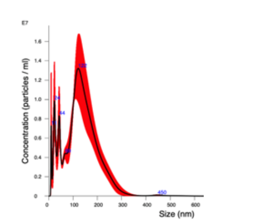	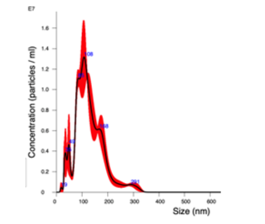
Mode	97.5 ± 36.4 nm (n=4)	86.2 ± 28.5 nm (n=7)	67.0 ± 37.8 nm (n=7)

Supplementary Figure 2: Comparison of different protocols and storage methods for BEV isolation from human stool samples

(A) Fecal EVs were isolated from fresh or frozen stool samples or from stool samples stored in DNA/RNA shield™ according to protocol II. Bar graph shows protein content of fEVs per gram stool isolated from fresh stool (light grey bars), from frozen stool (white bars) and from stool stored in DNA/RNA shield™ (dark grey bars) and after ultracentrifugation (empty bars) or precipitation (striped bars). (B+C) Bar graphs show the fEV size in nm (B) and the number of fEVs per gram stool (C) of fEVs isolated from frozen stool (white bars) or from stool stored in DNA/RNA shield™ (dark grey bars) and after ultracentrifugation (empty bars) or after precipitation (striped bars). (D) Representative histograms from NTA by NanoSight NS300.

Supplementary Figure 3

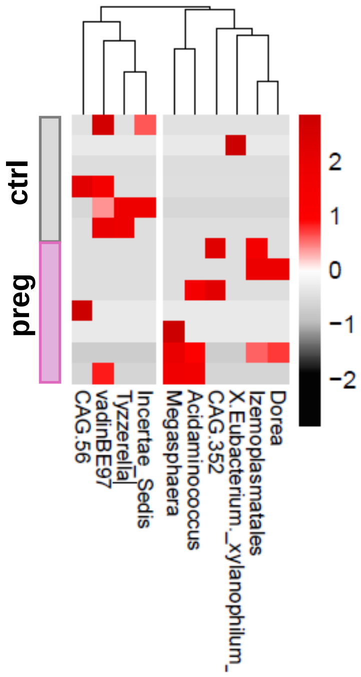

Supplementary Figure 3: Bacterial composition of fEV samples from non-pregnant controls and healthy pregnant women

Fecal EVs from non-pregnant controls and healthy pregnant women were analyzed by 16s rRNA sequencing. Heatmap shows differential expressed genera in fEV samples isolated from stool of control subjects and pregnant women. n=6-7

Supplementary Figure 4

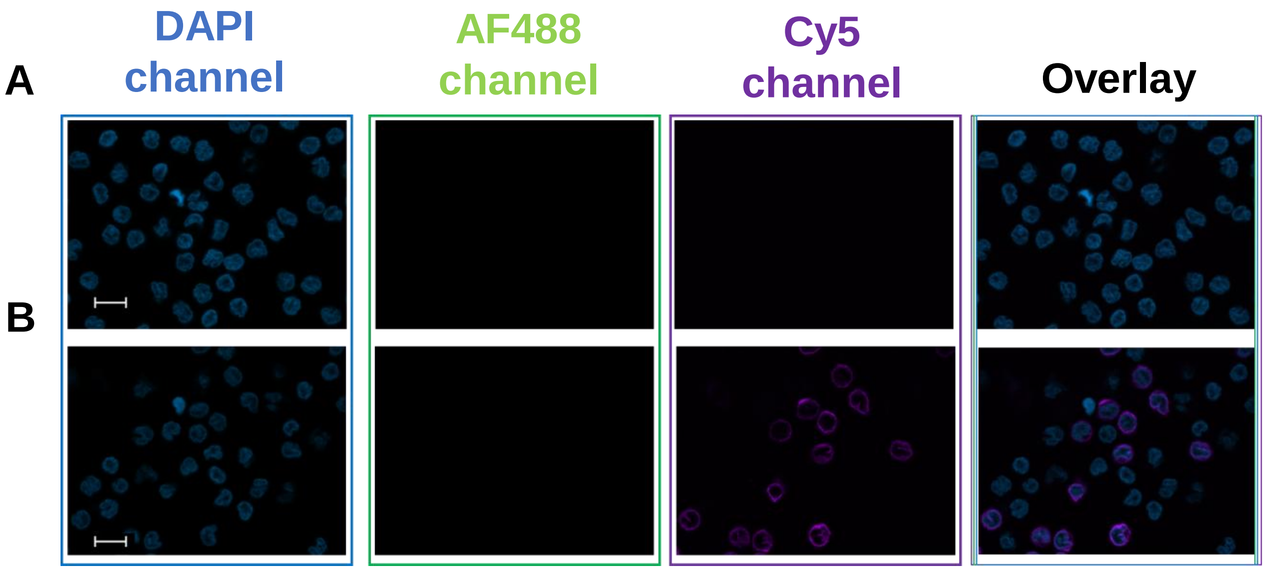

Supplementary Figure 4: Staining control for fluorescence microscopy of PBMC

To ascertain specific staining for CD3 and CFSE-stained fEVs, control staining of PBMCs was performed without incubating them with fEVs. Staining was performed in the presence and absence of the primary CD3 antibody, but with the secondary antibody coupled with AF647. The same settings as used in Figure 3A were used for the microscope incorporating the ApoTome system, including exposure time, and gamma value. Representative fluorescence microscopy images of PBMC incubated without CFSE-stained fEVs. (A) Nuclei were stained with Dapi (blue) and cells were treated with the secondary antibody for CD3 staining coupled with AF647 but not with the primary antibody against CD3. (B) Nuclei were stained with Dapi (blue) and T cells were immunostained with primary antibody against CD3 and the secondary antibody coupled with AF647 (violet, Cy5 channel). No signal was detected in the AF488 channel in which the CFSE-stained fEVs were visualized in Figure 3A. Magnification 100x; Scale bar 10 µm.

Supplementary Figure 5

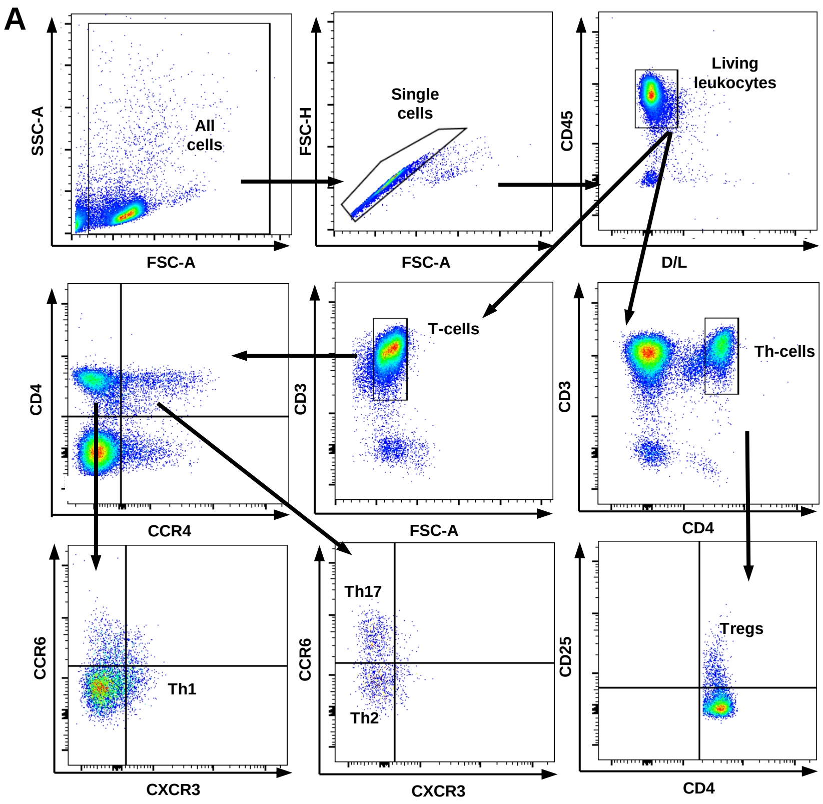

Supplementary Figure 5: Gating strategy for T cell subpopulations

Pseudocolor plots show gating strategy for $CCR4^-/CXCR3^+/CCR6^-$ T helper 1 cells, $CCR4^+/CCR6^-$ T helper 2, $CCR4^+/CCR6^+$ T helper 17 and $CD25^+$ regulatory T-cells.
