# Supplementary material for: Extracellular Vesicles Derived From the Feces of Pregnant Women Modulate T Cells Toward a Pregnancy‐Supportive Phenotype In Vitro
Source: Eur J Immunol. 2025 Sep 19;55(9):e70056. doi: 10.1002/eji.70056 (PMC12447864; doi:10.1002/eji.70056)
Supplement: Supplementary file 2 — Supporting file 2: eji70056‐sup‐0002‐Tables.docx. [file EJI-55-e70056-s002.docx]

**Supplementary Table 1**

|  | | control  (*n* = 14) | pregnant  (*n* = 24) |
| --- | --- | --- | --- |
| Age (years)^a^ | | 37.3 ± 5.2 | 35.3 ± 3.2 |
| gravidity^b^ | | 1.0 (2.0) | 2.0 (4.0) |
| parity^b^ | | 1.0 (2.0) | 1.0 (3.0) |
| Secondary diagnoses | | |  |
| Inflammatory | Allergies | 0 (0%) | 0 (0%) |
|  | Asthma | 1 (7%) | 0 (0%) |
|  | Inflammatory bowel disease (IBD) | 0 (0%) | 0 (0%) |
| Metabolic / Endocrine | Obesity | 0 (0%) | 0 (0%) |
|  | Diabetes | 0 (0%) | 0 (0%) |
|  | Hypothyroidism | 0 (0%) | 4 (17%) |
| Gynecological | Endometriosis | 0 (0%) | 1 (4%) |
|  | Polycystic ovary syndrome (PCOS) | 0 (0%) | 0 (0%) |

**Supplementary Table 2**

| protocol | Work steps |
| --- | --- |
| protocol I  Lajqi et al., 2022(63) | 1. centrifugation of diluted stool (1.500g, 10min) 2. centrifugation of the supernatant (10.000g, 10min) 3. filtration of the supernatant through 0.2µm filter |
| protocol II  Park et al., 2021(64) | 1. filtration of diluted stool through 100µm strainer 2. centrifugation of filtrated stool (10.000g, 10min) 3. filtration of the supernatant through 0.2µm filter 4. centrifugation (13.000rpm, 30min) |
| protocol III  Own, unpublished | 1. centrifugation of diluted stool/supernatant 3x (800g, 10min) 2. centrifugation of the supernatant 3x (10.000g, 10min) |
| protocol IV  Gao et al., 2018(65) | 1. centrifugation of diluted stool (200g, 10min) 2. centrifugation of the supernatant (4.500g, 30min) 3. filtration of the supernatant through 0.45µm filter 4. filtration of the supernatant through 0.2µm filter |
| protocol V  Park et al., 2018(66) | 1. centrifugation of diluted stool/supernatant 3x (800 g, 5min) 2. ultracentrifugation of the supernatant (100.000 g, 2h) 3. filtration of the supernatant through 0.45µm filter |
| protocol VI  Kang et al., 2013(67) | 1. centrifugation of diluted stool (10.000g, 20min) 2. filtration of the supernatant through 0.45µm filter 3. ultracentrifugation of the supernatant (100.000g, 2h, 4°C) |

**Supplementary Table 3**

| Protein name | Protein ID | p-value | Fold change ctrl/pre |
| --- | --- | --- | --- |
| NADH dehydrogenase [ubiquinone] 1 beta subcomplex subunit 4 | O95168 | 0.001 | -2.51 |
| Calpastatin | P20810-6 | 0.013 | -2.50 |
| T-cell differentiation antigen CD6 | P30203 | 0.044 | -2.43 |
| Heterogeneous nuclear ribonucleoprotein D-like | O14979 | 0.011 | -2.02 |
| Proteolipid protein 2 | Q04941 | 0.024 | -2.01 |
| Scaffold attachment factor B1 | Q15424 | 0.004 | -1.66 |
| 40S ribosomal protein S15 | P62841 | 0.038 | -1.61 |
| Cytosolic endo-beta-N-acetylglucosaminidase | Q8NFI3 | 0.022 | -1.53 |
| Vesicle-associated membrane protein-associated protein A | Q9P0L0 | 0.001 | -1.52 |
| N-acylneuraminate cytidylyltransferase | Q8NFW8 | 0.016 | -1.31 |
| Dermcidin;Survival-promoting peptide;DCD-1 | P81605 | 0.017 | -1.28 |
| Acetyltransferase component of pyruvate dehydrogenase complex | P10515 | 0.007 | -1.18 |
| Protein-L-isoaspartate O-methyltransferase | P22061 | 0.013 | -1.17 |
| S-phase kinase-associated protein 1 | E5RI56 | 0.042 | -1.02 |
| Nuclear ubiquitous casein and cyclin-dependent kinase substrate 1 | Q9H1E3 | 0.025 | -0.95 |
| V-type proton ATPase catalytic subunit A | P38606 | 0.016 | -0.93 |
| Nascent polypeptide-associated complex subunit alpha | E9PAV3 | 0.013 | -0.89 |
| Protein S100-A4 | P26447 | 0.013 | -0.77 |
| Glutathione S-transferase kappa 1 | Q9Y2Q3 | 0.008 | -0.75 |
| Phosphofurin acidic cluster sorting protein 1 | Q6VY07 | 0.020 | -0.71 |
| Bcl-2-associated transcription factor 1 | Q9NYF8 | 0.025 | -0.68 |
| Prelamin-A/C;Lamin-A/C | P02545 | 0.020 | -0.64 |
| Coatomer subunit beta | P35606 | 0.000 | -0.56 |
| Actin, cytoplasmic 1 | P60709 | 0.004 | -0.55 |
| FACT complex subunit SSRP1 | Q08945 | 0.012 | -0.53 |
| RNA-binding protein EWS | Q01844 | 0.022 | -0.53 |
| 40S ribosomal protein S3 | P23396 | 0.045 | -0.49 |
| Heterogeneous nuclear ribonucleoprotein L | P14866 | 0.024 | -0.40 |
| Leucyl-cystinyl aminopeptidase | Q9UIQ6 | 0.050 | -0.36 |
| WD repeat-containing protein 1 | O75083 | 0.028 | -0.33 |
| High mobility group protein B2 | P26583 | 0.008 | -0.28 |
| 40S ribosomal protein S16 | P62249 | 0.029 | -0.26 |
| Transaldolase | P37837 | 0.014 | -0.20 |
| Heat shock 70 kDa protein 6 | P17066 | 0.035 | -0.13 |
| T-complex protein 1 subunit zeta | P40227 | 0.006 | -0.11 |
| Heterogeneous nuclear ribonucleoprotein D0 | Q14103 | 0.042 | 0.15 |
| Leukotriene A-4 hydrolase | P09960 | 0.046 | 0.17 |
| Interferon-induced guanylate-binding protein 2 | P32456 | 0.032 | 0.22 |
| Plastin-2 | P13796 | 0.013 | 0.25 |
| Poly (rC)-binding protein 1 | Q15365 | 0.020 | 0.27 |
| Nucleosome assembly protein 1-like 4 | Q99733 | 0.035 | 0.32 |
| 60S ribosomal protein L7a | P62424 | 0.013 | 0.34 |
| ATP synthase subunit alpha, mitochondrial | P25705 | 0.035 | 0.35 |
| Carbonyl reductase [NADPH] 3 | O75828 | 0.046 | 0.38 |
| IST1 homolog | P53990 | 0.039 | 0.40 |
| Tubulin beta-4B chain | P68371 | 0.019 | 0.41 |
| Proliferation-associated protein 2G4 | Q9UQ80 | 0.049 | 0.41 |
| Low molecular weight phosphotyrosine protein phosphatase | P24666 | 0.042 | 0.44 |
| RuvB-like 1 | Q9Y265 | 0.048 | 0.46 |
| Dynactin subunit 1 | Q14203 | 0.035 | 0.52 |
| NADH-ubiquinone oxidoreductase chain 4 | P03905 | 0.002 | 0.53 |
| Cell division control protein 42 homolog | P60953 | 0.029 | 0.54 |
| Actin, alpha skeletal muscle | P68133 | 0.010 | 0.54 |
| Serine--tRNA ligase, cytoplasmic | P49591 | 0.019 | 0.57 |
| T-complex protein 1 subunit gamma | P49368 | 0.008 | 0.57 |
| Tubulin alpha-1A chain;Tubulin alpha-3E chain | Q71U36 | 0.047 | 0.59 |
| Mannose-P-dolichol utilization defect 1 protein | O75352 | 0.046 | 0.59 |
| Importin subunit alpha-5 | P52294 | 0.040 | 0.62 |
| Vasodilator-stimulated phosphoprotein | P50552 | 0.027 | 0.68 |
| Src kinase-associated phosphoprotein 1 | Q86WV1 | 0.039 | 0.81 |
| 60S ribosomal protein L14 | P50914 | 0.025 | 0.85 |
| Guanine nucleotide-binding protein subunit alpha-13 | Q14344 | 0.006 | 0.85 |
| Phosphatidylinositol transfer protein alpha isoform | Q00169 | 0.000 | 0.88 |
| Succinyl-CoA ligase [ADP-forming] subunit beta, mitochondrial | Q9P2R7 | 0.021 | 0.89 |
| ATP synthase subunit gamma, mitochondrial | P36542 | 0.007 | 1.02 |
| E3 ubiquitin-protein ligase RNF213 | Q63HN8 | 0.033 | 1.08 |
| Obg-like ATPase 1 | Q9NTK5 | 0.028 | 1.11 |
| Twinfilin-2 | Q6IBS0 | 0.041 | 1.11 |
| Glia maturation factor gamma | O60234 | 0.047 | 1.15 |
| Signal recognition particle receptor subunit beta | Q9Y5M8 | 0.001 | 1.24 |
| Translin-associated protein X | Q99598 | 0.014 | 1.28 |
| Heterogeneous nuclear ribonucleoprotein H2 | P55795 | 0.046 | 1.28 |
| Phosphoribosyl pyrophosphate synthase-associated protein 2 | O60256 | 0.032 | 1.28 |
| Nuclear pore complex protein Nup155 | O75694 | 0.029 | 1.31 |
| 26S proteasome non-ATPase regulatory subunit 12 | O00232 | 0.031 | 1.32 |
| Splicing factor U2AF 65 kDa subunit | P26368 | 0.012 | 1.37 |
| Retinol-binding protein 4 | P02753 | 0.015 | 1.53 |
| Signal recognition particle subunit SRP68 | Q9UHB9 | 0.028 | 1.57 |
| 60S ribosomal protein L4 | P36578 | 0.024 | 1.58 |
| Vacuolar protein sorting-associated protein 26A | O75436 | 0.027 | 1.65 |
| Enolase-phosphatase E1 | Q9UHY7 | 0.015 | 1.90 |
| CUGBP Elav-like family member 2 | O95319-5 | 0.022 | 1.98 |
| NADH dehydrogenase [ubiquinone] 1 beta subcomplex subunit 9 | Q9Y6M9 | 0.001 | 2.03 |
| Stromal membrane-associated protein 2 | Q8WU79 | 0.003 | 2.20 |
| Ras suppressor protein 1 | Q15404 | 0.028 | 2.44 |
| Crk-like protein | P46109 | 0.015 | 2.80 |
